# The Changes in the Frog Gut Microbiome and Its Putative Oxygen-Related Phenotypes Accompanying the Development of Gastrointestinal Complexity and Dietary Shift

**DOI:** 10.3389/fmicb.2020.00162

**Published:** 2020-03-03

**Authors:** Mengjie Zhang, Hua Chen, Lusha Liu, Liangliang Xu, Xungang Wang, Liming Chang, Qing Chang, Guoqing Lu, Jianping Jiang, Lifeng Zhu

**Affiliations:** ^1^Chengdu Institute of Biology, Chinese Academy of Sciences, Chengdu, China; ^2^School of Life Sciences, Nanjing Normal University, Nanjing, China; ^3^Hangzhou Legenomics Bio-Pham Technology Co., Ltd., Hangzhou, China; ^4^Department of Biology, University of Nebraska Omaha, Omaha, NE, United States

**Keywords:** amphibian metamorphosis, gastrointestinal remodeling, dietary shift, gut microbial composition and function, oxidative stress tolerance, chitinase expression

## Abstract

There are many examples of symbiotic and reciprocal relationships in ecological systems; animal gut microbiome–host interactions are one such kind of bidirectional and complex relationship. Here, we utilized several approaches (16S rRNA gene sequencing, metagenomics, and transcriptomics) to explore potential gut microbiome–host interactions accompanying the development of gastrointestinal complexity and a dietary shift from metamorphosis to maturity in ornamented pygmy frogs (*Microhyla fissipes*). We identified the possible coevolution between a particular gut microbial group (increased putative fat-digesting Erysipelotrichaceae and chitin-digesting *Bacteroides* and Ruminococcaceae) and the host dietary shift [from herbivore to insectivore (high proportion of dietary chitin and fat)] during metamorphosis. We also found that the remodeling and complexity of the gastrointestinal system during metamorphosis might have a profound effect on the gut microbial community (decreasing facultative anaerobic Proteobacteria and increasing anaerobic Firmicutes) and its putative oxygen-related phenotypes. Moreover, a high proportion of chitin-digesting bacteria and increased carbohydrate metabolism by gut microbiomes at the climax of metamorphosis would help the frog’s nutrition and energy needs during metamorphosis and development. Considering the increased expression of particular host genes (e.g., chitinase) in juvenile frogs, we speculate that host plays an important role in amphibian metamorphosis, and their symbiotic gut microbiome may help in this process by providing the nutrition and energy needs. We provide this basic information for the amphibian conservation and managements.

## Introduction

In ecology, there are many examples of symbiotic and reciprocal relationships (e.g., algae and marine invertebrates, mycorrhiza, gut microbes and animal hosts, and prokaryotes and protists or animals) (Smith and Douglas, 1987). Animal gut microbiome–host interactions play important roles in host nutrition, fitness, and health (Ley et al., 2008; Kinross et al., 2011; Zhu et al., 2011; Nicholson et al., 2012; McFall-Ngai et al., 2013; Wlodarska et al., 2015; Gould et al., 2018; Wei, 2018). The gut microbiota is involved in the regulation of multiple host metabolic pathways, giving rise to interactive host–microbiota metabolic, signaling, and immune-inflammatory axes (Nicholson et al., 2012). Additionally, the host, in turn, shapes the gut microbiome (Yatsunenko et al., 2012; Gould et al., 2018). In humans, the gut microbiome displays changes in composition and function in response to dietary changes during human development and physiological variations (Yatsunenko et al., 2012). For example, the abundance of *Bifidobacterium* (involved in human milk oligosaccharide degradation) decreases significantly from infancy to adulthood, and adults harbor different gut microbial communities (Yatsunenko et al., 2012). The changes in the gut microbial community under host development are a complex process involving many factors (e.g., gastrointestinal development, dietary changes, host genotype, geography, and environment) (Ley et al., 2008; Yatsunenko et al., 2012).

Metamorphosis is an extreme example of host development and is a biological process by which an animal physically develops after birth or hatching, involving a conspicuous and relatively abrupt change in the animal’s body structure through cell growth and differentiation (Dodd and Dodd, 1976; Denver, 2008). Some insects, fishes, amphibians, mollusks, crustaceans, cnidarians, echinoderms, and tunicates undergo metamorphoses (Truman and Riddiford, 1999; Laudet, 2011; Holstein and Laudet, 2014), which are often accompanied by a change in nutrient source or behavior (Dodd and Dodd, 1976; Denver, 2008). Some studies have explored the changes in gut microbiomes during metamorphosis in insects and fishes [*Heliconius erato* butterflies (Hammer et al., 2014), *Galleria mellonella* moth (Johnston and Rolff, 2015), *Spodoptera littoralis* leafworm (Chen et al., 2016), and sea lamprey *Petromyzon marinus* (Tetlock et al., 2012)] and have shown the extent of the influence of host nutritional resources or dietary shifts on microbial communities.

Metamorphosis in amphibians is accompanied by changes in morphology, physiology, and behavior (Wilbur and Collins, 1973; Werner, 1986; Newman, 1992; Shi, 2000). There are several key stages in metamorphosis, such as premetamorphosis (tadpoles), metamorphic climax (post-tadpoles), and completion of metamorphosis (frogs). Through metamorphosis, the frog undergoes the development of the limbs, gains the ability to breathe air using lungs, and may shift to a terrestrial lifestyle. The complexity of the gastrointestinal tract also develops between these life stages: from a simple and long gastrointestinal tract to a complex and complete digestive system (stomach, foregut, midgut, and terminal hindgut) (Hourdry et al., 1996; Schreiber et al., 2005). At the same time, many frog species complete the dietary shift during metamorphosis: from a plant material-based diet in tadpoles to primarily being insectivorous in adulthood (Jenssen, 1967; Linzey, 1967; Hendricks, 1973; Hourdry et al., 1996; Kupferberg, 1997; Castaneda et al., 2006). Several studies have investigated gut microbiota changes during metamorphosis in frogs [leopard frog *Lithobates pipiens* (Kohl et al., 2013), *Bufo gargarizans* (Chai et al., 2018), *Lithobates* [*Rana*] *sylvaticus* (Warne et al., 2017; Warne et al., 2019), *Lithobates clamitans* (Warne et al., 2017), and *Lithobates catesbeianus* (Warne et al., 2017)]. Kohl et al. (2013) found a significant difference (decreased Proteobacteria and increased Firmicutes) in the gut microbial community between tadpoles and frogs (mature) and suggested that measurements at various time points throughout metamorphosis will provide better insight into detailed gut microbial dynamics (Kohl et al., 2013). Chai et al. (2018) found shifts in microbial composition (e.g., a reduction in Proteobacteria and Actinobacteria) among five developmental stages from aquatic larvae to terrestrial juveniles (frog), but not mature adults (Chai et al., 2018). In their study, there were no significant changes in the relative abundance of Firmicutes compared to the previous research (Kohl et al., 2013), which may be caused by the different stages and species examined (Chai et al., 2018). These interesting studies reconstruct the gut microbial community at the composition level and provide information on the potential mechanism of gut microbiome–frog interactions during metamorphosis. In order to understand the gut microbiome development, future studies will need to integrate gut microbial functions and even host factors (e.g., gene expression) together with gastrointestinal complexity and dietary shift.

**FIGURE 1:**
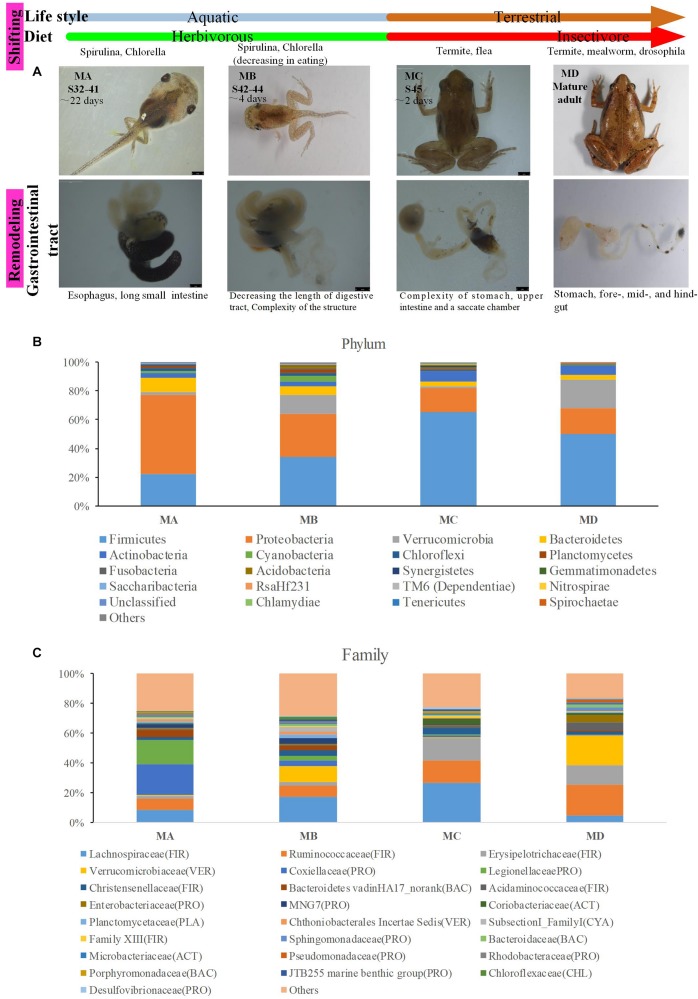
The gut microbiome changes from metamorphosis (herbivorous) to mature frogs (insectivorous). **(A)** The metamorphosis and remodeling of the digestive system among four groups (MA, stage 31–41; MB, stage 42–44; MC, stage 45; and MD, mature adults). **(B)** The domain phyla in the gut microbiome among groups using16S rRNA gene sequences. **(C)** The domain families in the gut microbiome among groups. The value in the *y* axis represents the mean relative abundance.

The ornamented pygmy frog *Microhyla fissipes* is a model for investigating the regulation of metamorphosis because of their smaller body size and shorter metamorphosis duration than those of *Xenopus*; 45 developmental stages have been defined in *M. fissipes* (Zhao et al., 2016; Wang et al., 2017). Stages 1 to 28 (early embryonic development period) cover fertilization to operculum completion (lasting for 82.6 h at 22–26.5°C). Stages 29 to 45 [larval (tadpole) development period] cover operculum completion to complete absorption of the tail (lasting for 38 days). Stages 32 to 41 [metamorphosis stage A (MA) in this study] cover the period starting from the knee junction of the hindlimb appearing (Figure 1A). Stages 42 to 44 [metamorphosis stage B (MB) in this study] cover the climax of metamorphosis, including forelimb development and tail resorption (Figure 1A). Stage 45 [metamorphosis stage C (MC) in this study] is the completed metamorphosis stage with complete tail absorption, and the frog shifts to an insect-based diet and mainly lives on the land (Figure 1A). Thus, from tadpole to frog [juveniles and mature adults [metamorphosis stage D (MD) in this study)], the ornamented pygmy frog displays several types of shift or remodeling, such as a dietary shift (from herbivore to insectivore), a lifestyle shift (from aquatic to terrestrial life), and a shift in the complexity of the gastrointestinal tract (acidic stomach, foregut, midgut, and terminal hindgut) (Figure 1A). Transcriptomic analysis of *M. fissipes* at different metamorphic stages identified genes that are likely to be involved in the regulation of metamorphosis (Zhao et al., 2016). Interestingly, Gene Ontology (GO) enrichment analysis at stage 45 showed that the majority of enriched GO categories were associated with carbohydrate metabolism (Zhao et al., 2016). This finding is consistent with feeding behavior (feeding resuming at the end of metamorphosis and putative and dietary shift). Thus, changes in diet, gastrointestinal physiology, and even host gene expression during metamorphosis make the ornamented pygmy frog a model to study gut microbiome–host interactions.

During metamorphosis in anurans, degeneration of the larval epithelium and the development of a new adult epithelium that is folded in the intestine occur (Hourdry et al., 1996; Ishizuyaoka, 2011). The folds in the intestine provide a vast surface area for absorption and aid in digestion (Fisher, 1955). The transport of fluid, nutrients, and electrolytes to and from the intestinal lumen is a primary function of epithelial cells. This process consumes large amounts of cellular energy and O_2_ (Ward et al., 2014). *In vitro* experiments also confirm that the germ-free mice luminal contents can chemically consume oxygen, such as via lipid oxidation reactions (Friedman et al., 2018). Thus, the development of gastrointestinal complexity (including the function of the acidic stomach and folding of the epithelium) may increase the consumption of O_2_ because of food fermentation by the symbiotic gut microbiota and the host themselves. Both the oxidative chemical reactions and the gut microbiome regulate luminal oxygen levels, shaping gut microbial composition throughout different regions of the intestine (He et al., 1999; Friedman et al., 2018).

Moreover, during metamorphosis, the endocrine cells of the digestive tract are also redistributed. Such changes [including the appearance of chitinases (EC: 3.2.14)] may be associated with modifications in feeding behavior (from herbivore to insectivore) (Hourdry et al., 1996). For example, chitin is a polymer of *N*-acetyl glucosamine and is a primary component of exoskeletons of arthropods (e.g., crustaceans and insects) (Kramer and Muthukrishnan, 1997; Doucet and Retnakaran, 2012). Chitin can be degraded by chitinases to generate GlcNAc (*N*-acetyl-D-glucosamine) and chito-oligosaccharides (Kramer and Muthukrishnan, 1997; Doucet and Retnakaran, 2012). Thus, here, we applied several approaches (16S rRNA gene sequencing, metagenomics, and transcriptomics of the host) to explore the potential gut microbiome dynamics in response to the gastrointestinal complexity and dietary shift from metamorphosis to mature adults including the following: (1) the putative dissimilarity in the gut microbial community throughout frog development; (2) the decrease in oxidative stress tolerance of gut microbiomes throughout metamorphosis; and (3) the potential adaptation to the insectivore diet (e.g., high proportion of chitin) in this symbiotic and reciprocal relationship.

## Results

### The Gastrointestinal Complexity and Dietary Changes From Metamorphosis to Mature Adults

**FIGURE 2:**
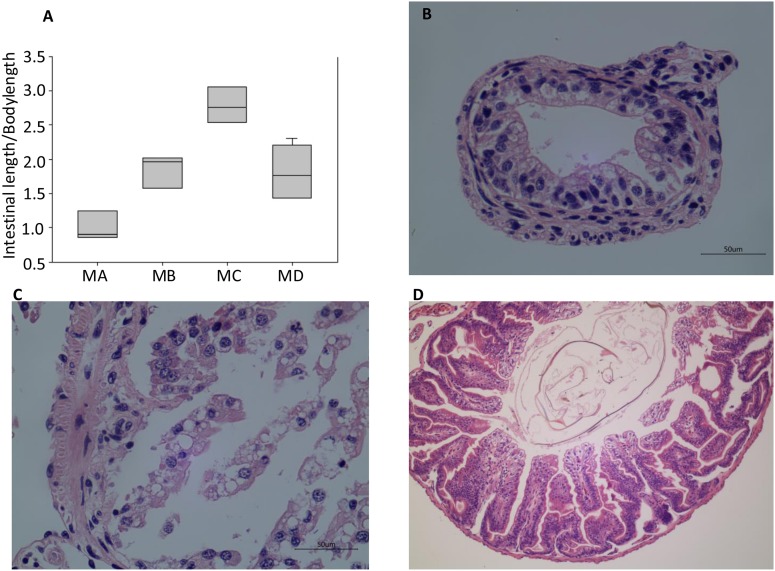
Morphological and histological observations of tadpole and frog intestines. **(A)** Cross sections of the intestine at four morphological stages. Intestinal fragments were cut approximately 8 cm from the stomach, which is the anterior of the small intestine. A significant difference was observed among the four groups (*df* = 3, *F* = 16.609, *p* < 0.001). Histological observations of MB **(B)**, MC **(C)**, and MD **(D)**. The black arrow shows the adipose tissue. There was no histological result from the MA stage because of a failed dissection in MA.

The digestive tract of tadpoles in group A (MA, S32-41) consists of the esophagus, stomach, and small and large intestines; the small intestine is long and is the most significant part of the gastrointestinal tract (Figure 1A). The ratio of the intestinal length to body length was lowest among the four stages. The stomach was only beginning to differentiate and could not be distinguished (Figure 1A). The chamber was full of digestive contents or juices mixed with algae. The primary diet was *Spirulina* and *Chlorella*. In group B (MB, S42-44), the intestine had undergone extensive changes. The length of the digestive tract was shorter (Figure 1A), whereas the upper and middle digestive tract expanded gradually (Figure 1A). The stomachs showed bulges compared to stomachs in group A. The inner wall of the intestinal tract was smooth, and there were few folds, whereas some surface absorption cells and goblet cells could be seen (Figure 2B). The primary diet was *Spirulina* and *Chlorella*, but organisms at this stage reduce eating.

In group C (MC, S45), metamorphosis had ended, tail degeneration was complete, and the tadpoles had become froglets (Figure 1A). The length of the entire gastrointestinal tract of the frogs was much shorter than that of tadpoles (Figure 1A). However, the ratio of the intestinal length to body length was highest among the four stages (Figure 2A). The upper gastrointestinal tract of the digestive tract was greatly expanded and appeared to be a saccade chamber, in which there was a visible protrusion. Histological observation showed that the epithelium had developed into the multiple-folded adult structure to increase the area of nutrient absorption (Figure 2C). In addition, the connective tissue and outer muscle were abundant and thickened. The amphibians shift from aquatic to terrestrial life. They also shift from a herbivore to an insectivore diet (e.g., termite and flea). In group D (MD, mature individuals), the gastrointestinal tract had finished intricate patterning, where the stomach, foregut, midgut, and terminal hindgut were discernible (Figure 1A). The intestinal wall epithelium was thicker than in previous stages and had more folds, and short rod-like villi were clearly visible. The lamina propria was also dense. There were a large number of goblet cells on the surface (Figure 2D). The major diet at this stage was mealworms and *Drosophila*.

### Changes in the Gut Microbial Community Similarity From Metamorphosis to Mature Adulthood

In this study, we gained the 18 pooled samples, and each pooled sample came from 10 individuals (Table 1). The Illumina HiSeq platform was used to generate the bacteria 16S rRNA sequences for these pooled samples. In order to avoid the bias in the sequencing depth, we rarefied our sequencing depth at ∼53,809 sequences per pooled sample based on the smallest sequencing depth in these pooled samples. The predominant phyla in the gut microbiota of the ornamented pygmy frogs in this study included Firmicutes, Proteobacteria, Verrucomicrobia, Bacteroidetes, Actinobacteria, and Cyanobacteria (Figure 1B). From metamorphosis to mature adults, the relative abundance of Firmicutes increased (MA: 22%, MB: 34%, MC: 65%, and MD: 50%), and the relative abundance of Proteobacteria (MA: 55%, MB: 30%, MC: 17%, and MD: 17%) and Bacteroidetes (MA: 10%, MB: 6%, MC: 4%, and MD: 3%) decreased. The relative abundance of Verrucomicrobia showed high variation among groups (MA: 2%, MB: 13%, MC: 1%, and MD: 20%). Linear discriminant analysis Effect Size (LEfSe) identified the gut microbial phyla with significantly differentiating abundance among groups (from metamorphosis to mature adults) including dominant Firmicutes (highest in MC), Proteobacteria (highest in MA), Chloroflexi (highest in MB), Cyanobacteria (highest in MB), Planctomycetes (highest in MB), and Fusobacteria (highest in MB) (Supplementary Figure S1).

**TABLE 1:** The top 10 microbial (genus level) contributions based on dissimilarity by SIMPER test.

MA vs. MB	Av. dissim	Mean MA	Mean MB	MA vs. MC	Av. dissim	Mean MA	Mean MC
Rickettsiella	6.492	0.133	0.00607	Legionella	7.017	0.148	0.00794
Legionella	6.043	0.148	0.0274	Rickettsiella	6.505	0.133	0.00684
Akkermansia	4.859	0.00632	0.102	[Anaerorhabdus] furcosa group	5.717	0.0103	0.125
Tyzzerella 3	3.941	0.0568	0.121	[Eubacterium] fissicatena group	5.004	0.00368	0.104
Aquicella	1.898	0.0699	0.034	Ruminococcaceae_uncultured	3.427	0.014	0.0825
Bacteroidetes vadinHA17_norank	1.799	0.0494	0.0313	Lachnospiraceae NK4A136 group	3.264	0.00333	0.0686
Saccharofermentans	1.479	0.0324	0.00283	Aquicella	3.179	0.0699	0.0063
Terrimicrobium	0.9923	0.0129	0.0217	Bacteroidetes vadinHA17_norank	2.407	0.0494	0.00123
Microcystis	0.9904	0.000637	0.0203	Tyzzerella 3	1.931	0.0568	0.0182
Ruminococcaceae_uncultured	0.9717	0.014	0.0334	Gordonibacter	1.707	0.00121	0.0354

**MA vs. MD**	**Av. dissim**	**Mean MA**	**Mean MD**	**MB vs. MC**	**Av. dissim**	**Mean MB**	**Mean MC**

Akkermansia	9.755	0.00632	0.2	[Anaerorhabdus] furcosa group	5.608	0.0125	0.125
Legionella	7.289	0.148	0.0025	Tyzzerella 3	5.138	0.121	0.0182
Rickettsiella	6.613	0.133	0.001	Akkermansia	4.889	0.102	0.00409
Ruminococcaceae_uncultured	6.427	0.014	0.142	[Eubacterium] fissicatena group	4.878	0.00621	0.104
Aquicella	3.41	0.0699	0.00167	Lachnospiraceae NK4A136 group	3.199	0.00523	0.0686
Acidaminococcaceae_uncultured	3.045	0.00729	0.0591	Ruminococcaceae_uncultured	2.455	0.0334	0.0825
Tyzzerella 3	2.497	0.0568	0.00685	Gordonibacter	1.669	0.00199	0.0354
Bacteroidetes vadinHA17_norank	2.444	0.0494	0.000494	MNG7_norank	1.618	0.037	0.00461
Salmonella	2.319	0.00459	0.048	Bacteroidetes vadinHA17_norank	1.502	0.0313	0.00123
[Anaerorhabdus] furcosa group	2.225	0.0103	0.0502	Aquicella	1.384	0.034	0.0063

**MB vs. MD**	**Av. dissim**	**Mean MB**	**Mean MD**	**MC vs. MD**	**Av. dissim**	**Mean MC**	**Mean MD**

Akkermansia	10.61	0.102	0.2	Akkermansia	9.822	0.00409	0.2
Tyzzerella 3	5.704	0.121	0.00685	Ruminococcaceae_uncultured	5.919	0.0825	0.142
Ruminococcaceae_uncultured	5.493	0.0334	0.142	[Eubacterium] fissicatena group	4.654	0.104	0.0107
Acidaminococcaceae_uncultured	2.934	0.00424	0.0591	[Anaerorhabdus] furcosa group	4.273	0.125	0.0502
Salmonella	2.325	0.00395	0.048	Lachnospiraceae NK4A136 group	3.304	0.0686	0.0032
[Anaerorhabdus] furcosa group	2.203	0.0125	0.0502	Acidaminococcaceae_uncultured	3.075	0.00882	0.0591
MNG7_norank	1.77	0.037	0.00157	Salmonella	2.329	0.00357	0.048
Aquicella	1.616	0.034	0.00167	Lachnospiraceae_uncultured	1.834	0.0422	0.00553
Bacteroidetes vadinHA17_norank	1.539	0.0313	0.000494	Gordonibacter	1.44	0.0354	0.00725
Faecalitalea	1.438	0.00386	0.0317	Faecalitalea	1.421	0.0091	0.0317

**FIGURE 3:**
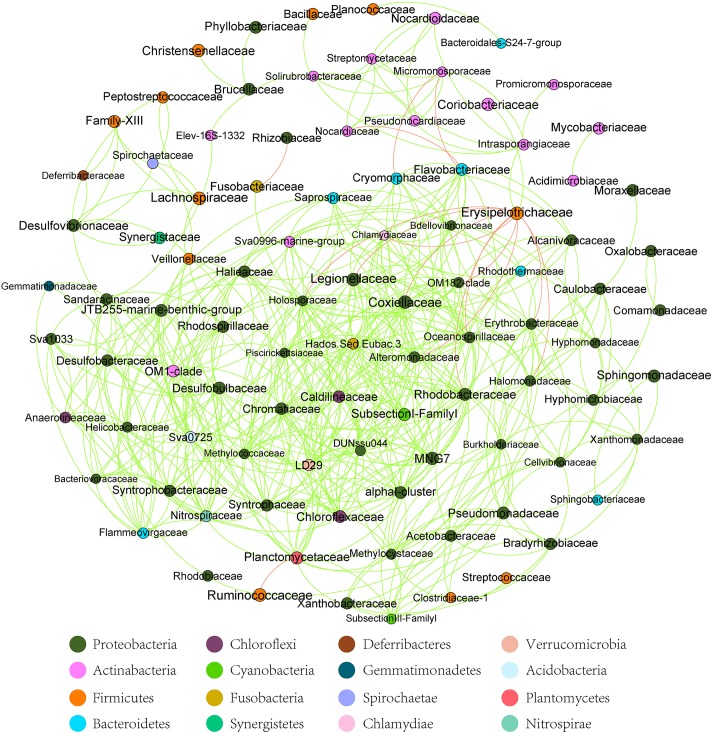
The co-occurrence analysis of the gut microbiome (family level) in all samples (with Spearman index ρ = 0.7). The dot represented the bacteria family. The red line between the dots represents the mutual exclusion relationship. The gray line between the dots represents the copresence relationship. The color of the dots represented the bacterial phylum. The red line represents the mutual exclusion; the green line represents the copresence.

**FIGURE 4:**
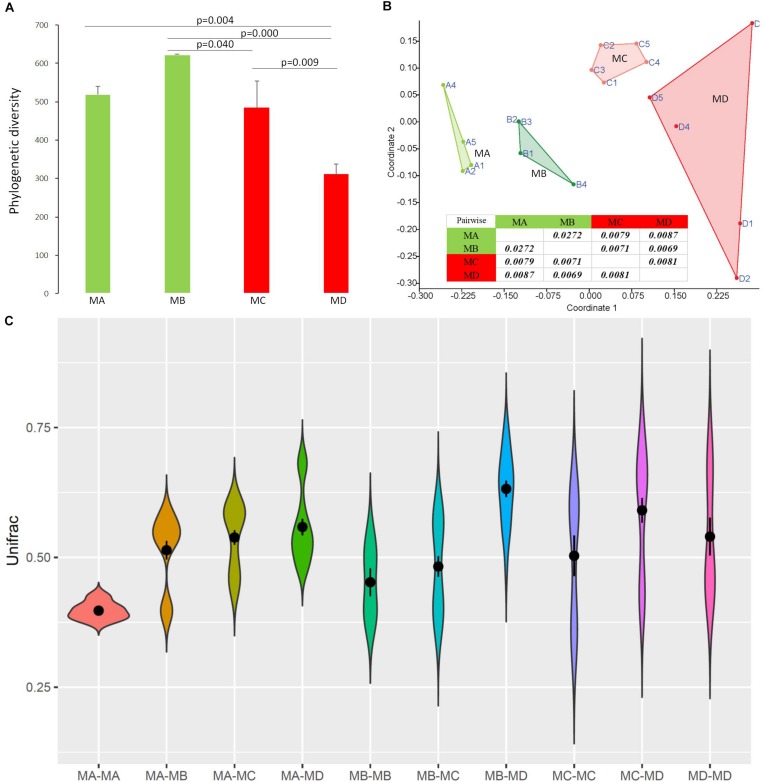
The diversity changes in the gut microbiome among groups using 16S rRNA gene sequences. **(A)** Phylogenetic diversity. **(B)** Bray-Curtis dissimilarities in the microbial composition (species abundance) among groups using NMDS (non-metric multidimensional scaling). The closure for each group was generated by Convex Hull (Barber et al., 1996). **(C)** The pairwise comparison was tested based on Bray-Curtis dissimilarities. The Unifrac unweighted distance among groups and within groups. Among groups: MA-MB, MA-MC, MA-MD, MB-MC, MB-MD, and MC-MD; within groups: MA-MA, MB-MB, MC-MC, and MD-MD. Because of the sample (pooled) variation in the gut microbiome composition, the distance within groups was over zero.

At the dominant family level (Figure 1C), the relative abundance of Ruminococcaceae (MA: 7%, MB: 8%, MC: 15%, and MD: 20%) and Erysipelotrichaceae (MA: 2%, MB: 2%, MC: 15%, and MD: 14%) increased from metamorphosis to mature adults. The relative abundance of Coxiellaceae (MA: 21 MB: 4, MC: 1%, and MD: 0.3) and Legionellaceae (MA: 16%, MB: 3%, MC: 1%, and MD: 0.3%) belonging to Proteobacteria decreased from metamorphosis to mature adults. The relative abundance of Lachnospiraceae (MA: 8%, MB: 17%, MC: 26%, and MD: 5%) and Verrucomicrobiaceae (MA: 1%, MB: 10%, MC: 0.4%, and MD: 20%) was highly variable from metamorphosis to mature adults. LEfSe identified the gut microbial family with significantly differentiating abundance among groups (from metamorphosis to mature adults), which were Lachnospiraceae (highest in MC), Erysipelotrichaceae (highest in MC), Coxiellaceae (highest in MA), and Legionellaceae (highest in MA) (Supplementary Figure S1). Additionally, the MA group harbored the significantly highest abundance of many families in the Proteobacteria among these four different development stages (from metamorphosis to mature adults) (Supplementary Figure S1). Moreover, co-occurrence analysis at the gut microbial family level detected 20 significant mutual-exclusion relationships, 14 of which occurred between Erysipelotrichaceae (Firmicutes) and the families from Proteobacteria (Figure 3). In addition, the phylogenetic diversity significantly decreased from metamorphosis to mature adults [one-way analysis of variance (ANOVA), *F* = 9.3, *p* = 0.001], and MD had the significantly lowest phylogenetic diversity (*post hoc*, least significant difference test at a significance level of 0.05) (Figure 4A).

Non-metric multidimensional scaling (NMDS) analysis showed gut microbial dissimilarity among groups (one-way permutational multivariate ANOVA: *F* = 4.2, *p* = 0.0001), and the gut microbiome of each group belonged to one different cluster. All of the pairwise comparisons among groups using Unifrac unweighted distance were significantly different (Figure 4B), and the pairwise distance showed that the gut microbial dissimilarity increased over the development stages to some extent (Figure 4C). For example, the Unifrac unweighted distance between MA and the other three groups or between MB and the other two groups (MC, MD) increased. The pairwise SIMPER test was used to further investigate the contribution of each gut microbial genus to the observed dissimilarity (Table 1). The greatest contribution to the dissimilarity between MA and each other group was caused by the highest abundance of the genera *Rickettsiella* (Proteobacteria_ Coxiellaceae) and *Legionella* (Proteobacteria_ Legionellaceae) in the MA groups. The greatest contribution to the dissimilarity between MB and each other group was caused by the high relative abundance of the genera *Akkermansia* (Verrucomicrobia_ Verrucomicrobiaceae) and *Tyzzerella 3* (Firmicutes_ Lachnospiraceae) in the MB groups. The greatest contribution to the dissimilarity between MC and each other group was caused by the highest abundance of the genera [*Anaerorhabdus*] *furcosa* group (Firmicutes_Erysipelotrichaceae) and [*Eubacterium*] *fissicatena* group (Firmicutes_ Lachnospiraceae) in the MC groups. The greatest contribution to the dissimilarity between MD and each other group was caused by the high abundance of genera *Akkermansia* (Verrucomicrobia_ Verrucomicrobiaceae) and *Ruminococcaceae_uncultured* (Firmicutes_ Ruminococcaceae) in the MD groups.

### Changes in the Putative Oxygen-Related Phenotypes of Gut Microbiome From Metamorphosis to Mature Adults

**FIGURE 5:**
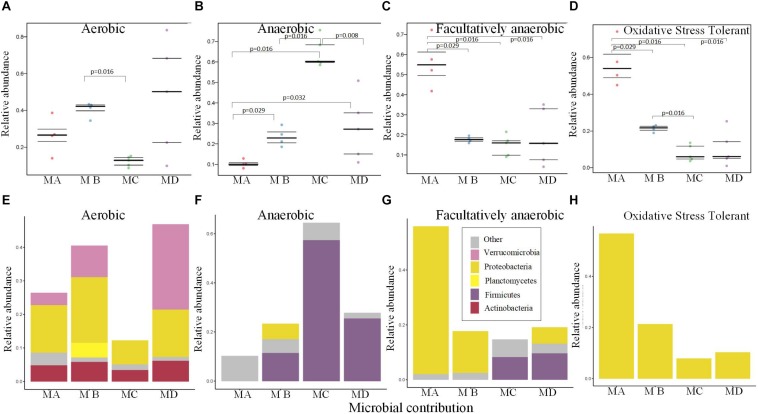
Bugbase (Ward et al., 2017) predicted the proportion of the bacteria involved in the oxygen-related phenotypes within the gut microbiome from metamorphosis (herbivorous) to maturity (insectivorous) in frogs. **(A)** The proportion of aerobic bacteria within microbiome of each sample. **(B)** The proportion of anaerobic bacteria within microbiome of each sample. **(C)** The proportion of facultatively anaerobic bacteria within microbiome of each sample. **(D)** The proportion of oxidative stress tolerance bacteria within microbiome of each sample. **(E)** The corresponding OTU contribution plots of the relative abundance of phyla related to aerobic phenotype. **(F)** The corresponding OTU contribution plots of the relative abundance of phyla related to anaerobic phenotype. **(G)** The corresponding OTU contribution plots of the relative abundance of phyla related to facultatively anaerobic phenotype. **(H)** The corresponding OTU contribution plots of the relative abundance of phyla related to oxidative stress tolerance phenotype.

The gut microbiome of MC had the lowest abundance of the aerobic bacteria (Figure 5A), which may be due to the low abundance of Proteobacteria and Verrucomicrobia (Figure 5E). The relative abundance of anaerobic bacteria increased from metamorphosis to mature adults; the lowest significant abundance was observed in MA, and the highest significant abundance was observed in MC (Figure 5B; pairwise Mann-Whitney-Wilcoxon test, *p* < 0.05). The changes in the abundance of Firmicutes mostly contributed to these variations (Figure 5F). The changes in the relative abundance of facultatively anaerobic and oxidative stress–tolerant bacteria decreased from metamorphosis to mature adults, and MA had the highest significant abundance (Figures 5C,D; pairwise Mann-Whitney-Wilcoxon test, *p* < 0.05). The changes in the abundance of Proteobacteria mostly contributed to these variations (Figures 5G,H).

### The Putative Function of the Frog Gut Microbiome From Metamorphosis to Mature Adults Using Metagenomics

**FIGURE 6:**
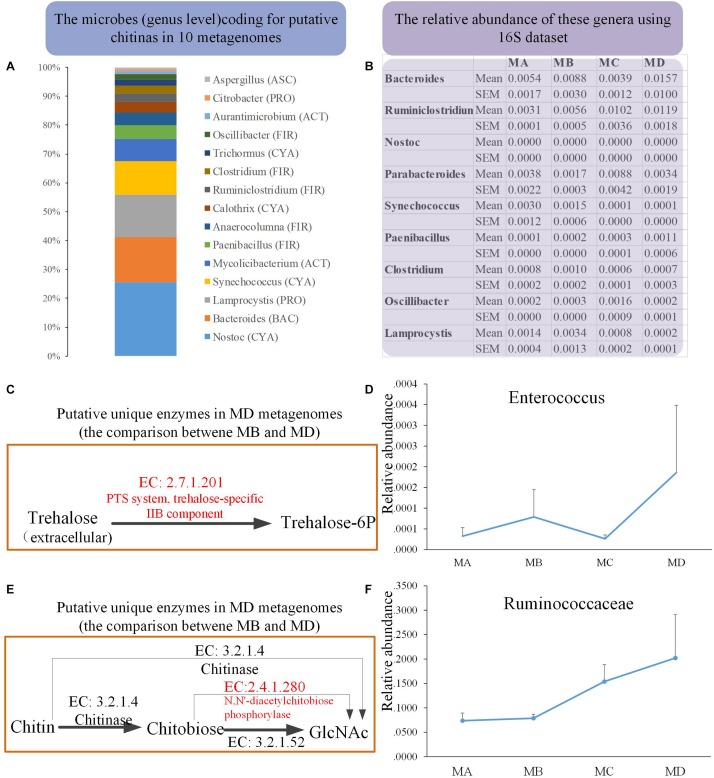
The metagenomics analysis of the MB and MD groups. **(A)** The taxon assignment (genus level) of genes coding for putative chitinases (EC 3.2.1.14). **(B)** The relative abundance of possible chitin-digesting bacteria using 16S rRNA gene sequences among groups. **(C)** The unique genes coding for the putative trehalose-specific IIB component [EC:2.7.1.201] in the MD group using metagenomics (comparing only the metagenomes between MB and MD) and the relative abundance of their origin (bacterial genus) using 16S data **(D)**. **(E)** The unique genes coding for putative *N,N*′-diacetylchitobiose phosphorylase [EC:2.4.1.280] in the MD group using metagenomics (comparing only the metagenomes between MB and MD) and the relative abundance of their origin (bacterial genus) using 16S rRNA gene sequences **(F)**.

We obtained 10 metagenomes (four from MB and six from MD) in 10 pooled gut content samples from 100 individuals. The major contaminants, especially in MD metagenomes, came from the host and the diet: Chordata (49.46%) and Arthropoda (25.43%) (Supplementary Tables S2, S3). Thus, after removing the contamination using three steps, the clean data set of prokaryote sequences for six metagenomes was very small (Supplementary Table S4) and would introduce bias compared with the four MB metagenomes (with the low contamination rate and the high proportion of clean data, mostly from prokaryotes). First, to reduce the bias caused by uneven data sets, we took one conservative strategy to investigate the unique genes detected in only most of the six MD genomes, none of which were identified in any MB metagenomes. Second, considering the dietary shift from herbivore to insectivore, we investigated the bacterial chitinases (EC: 3.2.1.14) degrading the chitin and their putative bacterial taxonomical assignment among these 10 clean metagenomes. The mean abundance of the gene coding for putative chitinase was 0.010 and 0.025% in MB and MD, respectively. Taxon assignment of these genes identified their putative bacterial origins, including genera *Bacteroidetes* (BAC), *Cyanobacteria* (CYA), *Firmicutes* (FIR), *Proteobacteria* (PRO) (Figure 6A). The 16S data revealed that most of these genera were rare in these four groups; only *Bacteroides* (1.57% in MD) and *Ruminiclostridium* (1.19%) had a relatively higher abundance in MD than in the other groups (Figure 6B).

Moreover, we also investigated whether the unique genes might code some putative enzymes involved in the degradation of chitin in MD metagenome after their dietary shift (from herbivore to insectivore) compared with the MB metagenome. Trehalose is the non-reducing disaccharide of glucose and is the principal sugar circulating in the blood or hemolymph of most insects (Thompson, 2003). According to the comparison in the metagenomes between MB and MD, three unique genes coding for putative trehalose-specific IIB component [EC: 2.7.1.201], *N,N*′-diacetylchitobiose phosphorylase [EC: 2.4.1.280], and histone-lysine *N*-methyltransferase SETMAR [EC: 2.1.1.43] were identified in the MD metagenomes (Figures 6C,E). The first two genes were successfully assigned to gained taxa. The genes coding for the putative trehalose-specific IIB component involved in transporting extracellular trehalose into the cell came from *Enterococcus devriesei* (Firmicutes_ Enterococcaceae). The 16S data revealed that the MD adult group had the highest mean abundance of *E. devriesei* among these groups (Figure 6D). The genes coding for the putative *N,N*′-diacetylchitobiose phosphorylase involved in chitin catabolism [catalyzing chitobiose to *N*-acetylglucosamine (GlcNAc)] came from Ruminococcaceae and *Ruthenibacterium lactatiformans* (Firmicutes_ Ruminococcaceae). The 16S data revealed that the relative abundance of Ruminococcaceae sharply increased from MC to MD, and MD samples had the highest mean abundance among these groups (Figure 6F). Thus, the metagenomes in the MD might have some features in response to the insectivorous diet.

### Gene Expression of Genes Coding for the Enzymes Involved in Chitin Metabolism During Metamorphosis Using Host Transcriptomics

**FIGURE 7:**
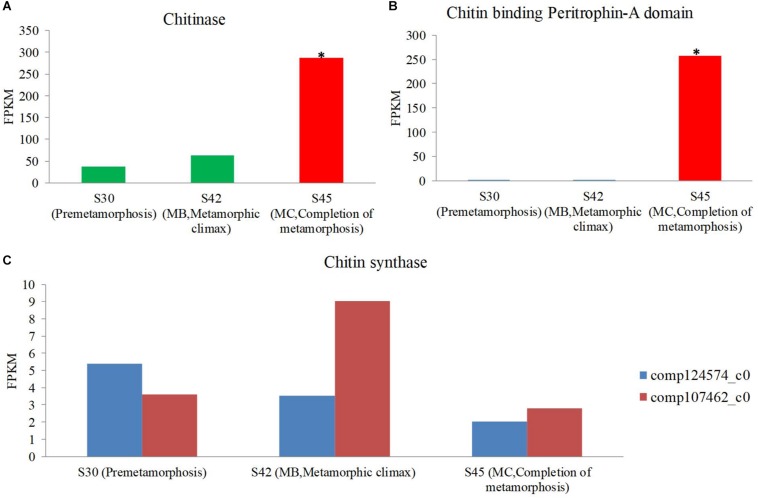
The expression level of the chitin metabolism gene using host transcriptomes among three stages [premetamorphosis (stage 30), metamorphic climax (stage 42, MB), and completion of metamorphosis (stage 45, MC)] (Zhao et al., 2016). **(A)** chitinase. **(B)** Chitin-binding peritrophin-A. **(C)** Chitin synthase.

One of the profound changes during metamorphosis is the dietary shift from herbivore to insectivore (herbivore: MA and MB, insectivore: MC and MD). The gene expression coding for the putative chitinase and chitin-binding peritrophin-A domain increased during metamorphosis, and the expression of this gene was most significant in the MC stage among the three stages [S30 (premetamorphosis), MB, and MC] (Figures 7A,B). However, the expression of genes coding for putative chitin synthase was rare, and there was no significant difference in expression level among these three stages. Thus, during the dietary shift, the juvenile frog in the MC stage eating insects had higher chitin-digesting gene expression level than tadpoles eating herbivorous food.

## Discussion

Significant changes in the gut microbiome and its related phenotypes were detected from metamorphosis to mature adult stages, which may reflect the putative relationship between the host and its symbiotic gut microbiome during the shift in diet and lifestyle and the remodeling of the gastrointestinal system.

The significant changes observed in the gut microbiome after a dietary shift were the increase in Firmicutes and the ratio of Firmicutes to *Bacteroides* in juvenile (MC) and mature frogs (MD) compared to tadpoles (MA and MB stages). The high proportion of Firmicutes and high ratio of Firmicutes to *Bacteroides* are the typical characteristics of the gut microbiomes of obese humans (Ley et al., 2005; Ley et al., 2006; Turnbaugh et al., 2006) and are associated with increased body weight (Verdam et al., 2013; Koliada et al., 2017). For example, the obese pmicrobiome has an increased capacity to harvest energy from the diet in humans and rats (Turnbaugh et al., 2006). Moreover, many studies have found that a high-fat diet leads to an increase in Erysipelotrichaceae in the gut (Turnbaugh et al., 2008; Turnbaugh et al., 2009; Daniel et al., 2014; Lecomte et al., 2015). There is a putative connection between Erysipelotrichaceae and host lipid metabolism, and a specific metabolic phenotype of the host (e.g., cholesterol excretion) may influence the gut microbiota (Martínez et al., 2012; Kaakoush, 2015). Thus, herein, the significant increase of Erysipelotrichaceae may be caused by the shift from to the herbivorous diet (detritivore) to the insectivorous diet; these bacteria were maintained throughout the frog metamorphosis stages (from juveniles to mature frogs) in this study.

The insect diet has a high proportion of chitin (Tang et al., 2015). Chitin, a long-chain polymer of *N*-acetylglucosamine, is a derivative of glucose. It is a primary component of cell walls in fungi; the exoskeletons of arthropods, such as crustaceans (e.g., crabs, lobsters, and shrimps) and insects; the radulae of mollusks; cephalopod beaks; and the scales of fish and lissamphibians (Tang et al., 2015). Here, we found that some bacteria (e.g., *Bacteroides* and Ruminococcaceae) with genes coding for putative chitin-digesting enzymes (e.g., chitinase and *N,N*′-diacetylchitobiose phosphorylase) increased during the dietary shift, and the mature frog had the highest abundance of these bacteria. At the host level, the juvenile frog (MC) was significantly enriched in genes coding for the putative chitinase and chitin-binding peritrophin-A domain compared to tadpoles. The juvenile and adult frogs are predators (e.g., insect eaters), and this new feeding behavior is also associated with the appearance of new enzymes (e.g., chitinase, trypsin, and pepsin) to digest meat or chitin (Hourdry et al., 1996). Gut chitinase expression is detected in the juvenile frogs (*Xenopus laevis* and *Rana catesbeiana*) when the frogs change from a herbivorous to an insect-enriched diet, which suggests that the expression of gut chitinase may be regulated to meet the demand for the enzyme to digest chitin-coated foods (Suzuki et al., 2002). Thus, given the increase of putative chitin-digesting bacteria and the associated gene expression during metamorphosis in this study, we suggest that both the host and the gut microbiome may play an important role in insectivorous dietary adaptation in frogs.

During metamorphosis, we found significant changes in the oxygen-related phenotypes of the gut microbiomes starting during MB, and MC had the lowest abundance of oxidative stress–tolerant bacteria and highest abundance of anaerobic bacteria, which are mostly due to the significant decrease in Proteobacteria and increase in anaerobic Firmicutes. Tadpoles have an aquatic lifestyle and have filter-feeding behavior. Proteobacteria are the main phylum in water. Among the most frequent bacterial groups in drinking water are members of the phylum Proteobacteria (Hoefel et al., 2005; Vaz-Moreira et al., 2017). Water has dissolved oxygen. The filter-feeding and single digestion system in tadpoles would increase the colonization of facultatively anaerobic bacteria, such as Proteobacteria. For example, *Legionella*, belonging to Proteobacteria, is common in many environments, including aquatic systems (Muraca et al., 1990; Yu-Sen Lin et al., 1998). *Rickettsiella*, belonging to Proteobacteria, is also found in water and many aquatic invertebrates (e.g., crustaceans) (Friedman et al., 1997; Küchler et al., 2009). In this study, both *Legionella* and *Rickettsiella* were significantly highest in abundance in MA than MB, MC, or MD, where they were rare. In the MB stage (aquatic lifestyle), the diet is the same as the MA stage. However, the climax of metamorphosis, including the increased complexity of the digestive system, requires energy for development. At this stage, the tadpoles do not eat or eat less, and most of the energy for the metamorphosis or development comes from the degeneration of tissues and obtained by oxidation of accumulated reserves (e.g., fat and carbohydrate oxidation) (Hourdry et al., 1996; Warne et al., 2017); these metabolic reactions will consume oxygen in the body. Thus, the increased complexity of the digestive system may lead to a decrease in these facultatively anaerobic bacteria and an increase in anaerobic bacteria (e.g., Firmicutes).

Moreover, MC (terrestrial lifestyle) is the completion of metamorphosis, including the complete digestive system. In this step, most metabolic reactions by the host [e.g., degeneration of tails, fat, and carbohydrate (e.g., glycogen) oxidation] and fermentation by gut microbes continue to consume oxygen. Interestingly, GO enrichment analysis at the MC stage (stage 45) showed that the majority of enriched GO categories were associated with carbohydrate metabolism (e.g., glycolysis, gluconeogenesis, hexose biosynthetic process, monosaccharide biosynthetic process, glucose catabolic process, hexose catabolic process, monosaccharide catabolic process, glucose metabolic process, single-organism carbohydrate catabolic process, carbohydrate catabolic process, and carbohydrate biosynthetic process) (Zhao et al., 2016). Gut microbial function in the MC stage had the highest abundance in bacteria associated with carbohydrate metabolism among these four stages (Supplementary Figure S2). In addition, the new enzymes of the host related to food digestion and complexity of the digestive system (acidic stomach and folded epithelium of intestine) further increase food fermentation and oxygen consumption in the gastrointestinal system. Thus, all these factors may contribute to form the anaerobic habitat for the juvenile frog’s digestive system in the MC stage and increase the colonization of anaerobic gut microbes and the selection of particular gut microbial groups together with the dietary shift. Considering the effect on the gut microbiome composition by luminal oxygen levels (He et al., 1999; Friedman et al., 2018), there is another possible example of the relationship between the host and the gut microbiome: the high carbohydrate metabolism level of gut microbiomes in the MC stage may provide energy for host tissue rebuilding in addition to the energy provided by the host.

Additionally, in the MD (adult) stages, we found that the proportion of anaerobic bacteria decreased compared to the MC stage. A carnivorous animal has a relatively simple and short digestive system (Hume, 2002). The adult frog has a large food intake (from large insects) compared to the juvenile frog in the MC stages. Thus, the MD stage will increase the change of colonization by aerobic environmental bacteria, such as Verrucomicrobia, acquired from their new living conditions and even from their new diet. Verrucomicrobia is one of the dominant bacteria in the environment. The decrease in alpha diversity of the gut microbiome from metamorphosis (herbivorous) to juvenile and mature frogs (insect-enriched) has also been detected between herbivorous and carnivorous vertebrates (Ley et al., 2008).

## Conclusion

Here, we identified some putative relationships between the frog and its gut microbiome. (1) We revealed the gut microbiome developmental association that was influenced by host dietary sources. (2) The remodeling of the gastrointestinal system during metamorphosis might also have a profound effect on the gut microbial composition. (3) The potential functions of the gut microbiome could help the frog’s nutritional and energy needs during metamorphosis and development. Therefore, these findings provide the basic information for amphibian management and conservation.

## Materials and Methods

### Sample Collection and Gut Content Preparation

A total of 80 individual tadpoles and 160 individual frogs (Supplementary Table S1) were collected from the habitat around the Wild Research Center of the Chengdu Institute of Biology (E: 104°9′12, N: 31°6′35) located in Shifang County in Sichuan Province from June to August 2017. Based on our previous experience on the feeding and development of *M. fissipes* in the captive environment and direct observation in the wild field [e.g., Zhao et al. (2016)], we could identify these tadpoles from *M. fissipes* and estimate the primary diet of *M. fissipes*. The tadpoles were collected gently with fishing nets, and the young and adult individuals were captured after metamorphosis at night with sterile gloves (usually 21:00 to 24:00 is the active period). To ensure the integrity of the contents in the digestive tract, the frogs were immediately euthanized with MS-222. Tadpoles were treated at a concentration of 0.3 to 0.6 g/L MS-222 for 2 to 3 min, and frogs were treated for 5 to 8 min or more. After washing the surface of the animals repeatedly with sterile distilled water to avoid residual solution, we removed the holonomic gastrointestinal tract from the abdominal cavity and scraped the inner wall of the intestines with sterile blades or squeezed the intestines directly by hand. All of the gut contents or digestive juices were placed into 2-mL centrifugal tubes and then stored at −80°C until DNA extraction. Because of the lack of enough gastrointestinal content in a single tadpole, subadult or even adult individuals, we refer to the mixed treatments as the northern leopard tadpoles and frogs (Kohl et al., 2013). In our study, four typical stages of growth and developmental process (Shimizu-Nishikawa et al., 2003; Wang et al., 2017) were selected: before forelimb and after hindlimb growth (S32–41), marked as group MA; during the peak of metamorphosis and when the forelimb began to grow (S42–44), labeled as group MB; frogs that had recently completed metamorphosis (S45), labeled as group MC; and sexually mature individuals, marked as group MD. In total, we obtained 18 qualified samples of intestinal contents or digestive juices, including four tubes in group MA, four tubes in group MB, five tubes in group MC, and five tubes in group MD. Ten tadpole individuals were pooled for each tube (Table 1).

### Morphological and Histological Observations of Tadpole and Frog Intestines

We measured the mean ratio of the intestinal length to body length among four groups (MA, 3; MB, 3; MC, 3; and MD, 5 individuals). A stereo microscope (Nanjing Jiangnan Yongxin JSZ8 Stereo Microscope, Nanjing, China) with an MShot Digital Imaging System (microscope camera Mc50-N, Guangzhou, China) was used to observe, take photos of tadpoles, and measure their body length and intestinal length. Then, the small intestines were dissected and 4% paraformaldehyde fixed. After dehydration in a graded series of ethanol and transparency by xylene, intestines were embedded in paraffin and sectioned in serial transverse sections (4 μm thick) using an RM2016 [Leica RM2016 Manual Rotary Microtome (Wetzlar, Germany); Leica Microsystems]. Dewaxed serial sections were stained with Delafield’s hematoxylin and counterstained with eosin to show general histological characteristics.

### DNA Extraction and Bacterial 16S rRNA Sequencing

Gastrointestinal samples were thawed on ice, and microbial genomic DNA was extracted using a QIAamp Fast DNA Stool Mini Kit (QIAGEN, Hilden, Germany) according to the manufacturer’s protocol. The integrity of the DNA was visually assessed using 1.0% agarose gel electrophoresis and quantified using a Qubit and NanoDrop. The highly variable V4 region of the 16S rRNA gene was amplified from community genomic DNA using the bacteria-specific universal primers 515F (GTGCCAGCMGCCGCGGTAA) and 806R (GGACTACHVGGGTWTCTAAT). Polymerase chain reaction was performed in triplicate using a 25 μL reaction containing ∼40 ng of DNA template, 2.5 μL of 10 × TransStart Taq buffer, 1 μL of each forward and reverse primer, 2 μL of dNTPs (2.5 mM), 0.25 μL of TransStart Taq DNA Polymerase, and 16.25 μL of ddH_2_O. The polymerase chain reaction amplification conditions were as follows: initial denaturation at 94°C for 5 min, followed by 35 cycles of denaturation at 94°C for 30 s, annealing at 53°C for 30 s, elongation at 72°C for 30 s, and a final extension at 72°C for 10 min. Polymerase chain reaction products were purified with a Universal DNA Purification Kit (TIANGEN, Beijing, China), and barcoded V4 amplicons were sequenced using the Illumina HiSeq platform (HiSeq2500 PE250).

### Data Analysis

Raw sequences were generated from the Illumina HiSeq sequencing platform. We performed quality control (e.g., demultiplexing and denoising) and taxon classification (based on Silva 132 version) in QIIME1.9 (Caporaso et al., 2010). We obtained the operational taxonomic unit (OTU) abundance tables and diversity results for downstream analysis. We chose to rarefy our sequencing depth at ∼53,809 (according to the lowest number of sequences of one sample in this study) to equalize the sampling depth across all samples.

#### Gut Microbial Community Analysis

We used LEfSe (Segata et al., 2011) to determine the gut microbial taxon with significantly differentiating abundance among groups (development stages). The relative abundance table of the bacteria families was inputted into Cytoscape 3.4.0 (Shannon et al., 2003). And then, we used the plugin CoNet (Faust and Raes, 2016) to generate co-occurrence plots using these parameters (Spearman index, ρ = 0.7). CoNet can detect significant non-random patterns of co-occurrence (mutual exclusion and copresence) in abundance and incidence data (Faust and Raes, 2016). The alpha diversity (e.g., phylogenetic diversity) among groups was analyzed using one-way ANOVA in SPSS Statistics 20.0 (IBM SPSS, 2011).

#### Gut Microbial Beta Diversity Analysis

The Bray-Curtis distance for species abundance was used to generate NMDS in PAST3 (Hammer et al., 2001). Moreover, to evaluate the effect of development stages on the gut microbiota composition, we performed one-way permutational multivariate ANOVA on Bray-Curtis dissimilarities in species abundance in PAST3 (Hammer et al., 2001). To determine the dissimilarity trend over the development stages, we performed pairwise comparisons among groups on Unifrac unweighted distance (Lozupone et al., 2011). The contribution of each gut microbial genus to the observed dissimilarity between groups was performed by Similarity Percentages test (SIMPER) in PAST3 (Hammer et al., 2001).

#### Microbial Phenotype Prediction

We used BugBase to predict the oxygen-related phenotypes of the frog gut microbiome (Ward et al., 2017). BugBase is an algorithm that predicts organism-level coverage of functional pathways as well as biologically interpretable phenotypes such as oxygen tolerance and Gram staining within complex microbiomes using either marker gene sequencing data (e.g., 16S) or whole-genome shotgun data (Ward et al., 2017). For example, reference databases in BugBase included Integrated Microbial Genomes (Markowitz et al., 2011), the Kyoto Encyclopedia of Genes and Genomes (KEGG) (Kanehisa et al., 2011), and the Pathosystems Resource Integration Center (Snyder et al., 2006), which were categorized to six major phenotypes (e.g., Gram staining, oxygen tolerance, ability to form biofilms, mobile element content, pathogenicity, and oxidative stress tolerance) (Ward et al., 2017). Here, the OTU table of 16S data was normalized by 16S copy number and then was input into the BugBase to gain the trait prediction by algorithm methods based on the reference databases (Ward et al., 2017). Pairwise Mann-Whitney-Wilcoxon tests were performed among groups.

### Metagenomic Sequencing and Data Analysis

#### Metagenomic Sequencing

Metagenomic shotgun sequencing libraries were prepared and sequenced at Shanghai Biozeron Biological Technology Co. For each sample, 1 μg of genomic DNA was used with Illumina’s TruSeq for library preparation. Libraries were sequenced using the Illumina HiSeq 4000, PE 150.

#### Removal of Putative Host and Diet Contamination

HiSeq reads were filtered using custom Perl scripts and Trimmomatic (parameters: Trimmomatic-0.30.jar PE -phred33 LEADING:0 TRAILING:20 SLIDINGWINDOW:50:20 MINLEN:50) (Bolger et al., 2014) to remove (i) all reads less than 50 bp in length, (ii) reads with degenerate bases (N′s), and (iii) all duplicates defined as sequences whose initial 20 nucleotides were identical and shared an overall identity of greater than 97% throughout the length of the shortest read. After blasting with the NR databases using diamond (Buchfink et al., 2014), we removed the putative host (frog) and diet (insect) contamination and gained clean reads. This was the first step to remove putative host contamination. Megahit (Li et al., 2015) was used to assemble the clean reads (removing the contigs with coverage <60%), and prodigal was used for gene prediction (Hyatt et al., 2010). Then, we blasted these genes against the NR database in the National Center for Biotechnology Information using diamond and gained the putative taxon assignments of these genes per metagenome (Buchfink et al., 2014). Thus, we classified the taxon information for these genes into three categories, such as prokaryotes, and the different contamination sources (e.g., host, diet). Then, we used salmon (Patro et al., 2015) to map the clean reads to these genes per metagenome and kept only the reads theoretically belonging to prokaryotes. This was the second step in removing the host or diet (insect) contamination per metagenome. We used megahit (Li et al., 2015) to assemble these clean reads into contigs and remove the contigs with coverage of less than 60%. Prodigal (Hyatt et al., 2010) was used for gene prediction from these high-quality (flited) contigs, and we gained the gene files in this step. CD-HIT (Li and Godzik, 2006) was used to construct non-redundant gene sets with less than 90% overlap and less than 95% shared sequence identity from these gene files. We used diamond (Buchfink et al., 2014) to conduct species annotation for the non-redundant gene profile, remove the genes putatively belonging to the host and diet, and gain the final clean non-redundant gene profile. This was the final step to remove contamination. The referred published frog genome is not well assembled, which lead to the poor blasting for some metagenome reads. Thus, we used this third step to remove the putative contaminations based on non-redundant gene sets.

#### Clean Read Processing and KEGG Analysis

Based on these gene profiles, we used salmon (Patro et al., 2015) to map the clean reads (from step 2 after removing contamination) per metagenome to the clean non-redundant gene profile and determine the TPM abundance (transcripts per million reads) of these non-redundant gene profiles in each metagenome. Finally, the clean non-redundant gene sequences were searched against the KEGG database using diamond (Buchfink et al., 2014). The KEGG orthology, enzyme commission, and KEGG pathways associated with each sequence were determined. We calculated the relative abundance of KEGG pathways using TPM (transcripts per million) (Wagner et al., 2012).

### Transcriptome Analysis

We reanalyzed the transcriptome data in our previously published study (Zhao et al., 2016). These transcriptomes represented the three key developmental stages of *M. fissipes*: premetamorphosis (stage 30), metamorphic climax (stage 42, MB), and completion of metamorphosis (stage 45, MC). Three individuals from each of the three stages were used as independent biological replicates. Here, we focused on the genes coding for putative enzymes involved in the chitin metabolic pathway due to the dietary changes in these frogs. To compare the unigene expression levels, each unigene was further normalized by fragments per kilobase of exon model per million mapped reads for the three developmental stages (Trapnell et al., 2010). The transcriptome analysis focused on the changes in gene expression during metamorphosis and did not include the adult stage samples.

## Author’s Note

This study has also used the previous published transcriptome data (Zhao et al., 2016) with accession number (GECV00000000) at NCBI.

## Data Availability Statement

The 16S sequencing data and mapfile have been deposited in figshare (10.6084/m9.figshare.11627460). The raw metagenome data is being uploaded to NCBI (National Center for Biotechnology Information) with the accession number PRJNA601807. The requirement of the raw data and materials in this study can also send to LZ (zhulf@ioz.ac.cn).

## Ethics Statement

The animal use protocol in this study (permit: 2015-AR-JJP-01) was reviewed and approved by the Animal Ethical and Welfare Committee of Chengdu Institute of Biology, Chinese Academy of Sciences, Chengdu, China. The Chairman of this committee is Dr. Xinquan Zhao.

## Author Contributions

LZ and JJ conceived the project. MZ, LX, XW, and LC collected the samples. MZ performed the experiments. LZ, MZ, HC, LL, and GL analyzed the data. MZ, QC, LZ, JJ, and LG wrote the manuscript. All authors gave final approval for publication.

## Conflict of Interest

HC was employed by Hangzhou Legenomics Bio-Pham Technology Co., Ltd. The remaining authors declare that the research was conducted in the absence of any commercial or financial relationships that could be construed as a potential conflict of interest.
