# Supplementary material for: The Changes in the Frog Gut Microbiome and Its Putative Oxygen-Related Phenotypes Accompanying the Development of Gastrointestinal Complexity and Dietary Shift
Source: Front Microbiol. 2020 Mar 3;11:162. doi: 10.3389/fmicb.2020.00162 (PMC7062639; doi:10.3389/fmicb.2020.00162)
Supplement: Supplementary file 1 [file Data_Sheet_1.pdf]

Supplementary Material

Table S1 The sample information in this study

SampleID	Stage	Pooling	Diet
**16S miseq**			
A1	S32-41	a01-a10	Spirulina, Chlorella
A2	S32-41	a11-a20	Spirulina, Chlorella
A4	S32-41	a31-a40	Spirulina, Chlorella
A5	S32-41	a41-a50	Spirulina, Chlorella (eating less)
B1	S42-44	b01-b10	Spirulina, Chlorella (eating less)
B2	S42-44	b11-b20	Spirulina, Chlorella (eating less)
B3	S42-44	b21-b30	Spirulina, Chlorella (eating less)
B4	S42-44	b31-b40	Spirulina, Chlorella (eating less)
C1	S45	c01-c10	Termite,flea
C2	S45	c11-c20	Termite,flea
C3	S45	c21-c30	Termite,flea
C4	S45	c31-c40	Termite,flea
C5	S45	c41-c50	Termite,flea
D1	Mature adults	d01-d10	mealworm, drosophila
D2	Mature adults	d11-d20	mealworm, drosophila
D3	Mature adults	d21-d30	mealworm, drosophila
D4	Mature adults	d31-d40	mealworm, drosophila
D5	Mature adults	d41-d50	mealworm, drosophila
**Metagenome**			
B1	S42-44	b01-b10	Spirulina, Chlorella (eating less)
B2	S42-44	b11-b20	Spirulina, Chlorella (eating less)
B3	S42-44	b21-b30	Spirulina, Chlorella (eating less)
B4	S42-44	b31-b40	Spirulina, Chlorella (eating less)
D1	Mature adults	d51-d60	mealworm, drosophila
D2	Mature adults	d61-d70	mealworm, drosophila
D3	Mature adults	d71-d80	mealworm, drosophila
D4	Mature adults	d81-d90	mealworm, drosophila
D5	Mature adults	d91-d100	mealworm, drosophila
D6	Mature adults	D101-d110	mealworm, drosophila

Table S2 Metagenomic information and the proportion of contamination in these raw reads

#SampleID	RawData	CleanData	Rate	Contamination rate
D1	65932692	51871536	78.67%	21.33%
D2	64816092	51160618	78.93%	21.07%
D3	68302524	53963456	79.01%	20.99%
D4	72490712	56368142	77.76%	22.24%
D5	53265218	42246164	79.31%	20.69%
D6	41372386	32778698	79.23%	20.77%
B1	88046682	88001254	99.95%	0.05%
B2	74260812	74065882	99.74%	0.26%
B3	80113906	80064686	99.94%	0.06%
B4	82475962	82397396	99.90%	0.10%

Table S3 The taxon assignment of the contaminated raw reads in the total metagenomes

Taxon assignment (Phylum)	Proportion
d__Eukaryota;p__Chordata	49.46%
d__Eukaryota;p__Arthropoda	25.43%
d__Eukaryota;p__Chlorophyta	6.98%
d__Eukaryota;norank	6.72%
d__Eukaryota;p__Streptophyta	3.29%
d__Eukaryota;p__Cryptomycota	1.81%
d__Eukaryota;p__Ascomycota	0.94%
d__Eukaryota;p__Basidiomycota	0.74%
d__Eukaryota;p__Microsporidia	0.67%
d__Eukaryota;p__Mucoromycota	0.66%
d__Eukaryota;p__Cnidaria	0.53%
d__Eukaryota;p__Chytridiomycota	0.51%
d__Eukaryota;p__Nematoda	0.45%
d__Eukaryota;p__Zoopagomycota	0.42%
d__Eukaryota;p__Mollusca	0.38%
d__Eukaryota;p__Blastocladiomycota	0.23%
d__Eukaryota;p__Echinodermata	0.19%
d__Eukaryota;p__Platyhelminthes	0.11%
d__Eukaryota;p__Euglenida	0.08%
d__Eukaryota;p__Porifera	0.08%
d__Eukaryota;p__Priapulida	0.05%
d__Eukaryota;p__Bacillariophyta	0.05%
d__Eukaryota;p__Apicomplexa	0.05%
d__Eukaryota;p__Annelida	0.05%
d__Eukaryota;p__Tardigrada	0.04%
d__Eukaryota;p__Hemichordata	0.04%

d__Eukaryota;p__Brachiopoda	0.03%
d__Eukaryota;p__Placozoa	0.01%
d__Eukaryota;p__Rotifera	0.01%
d__Eukaryota;p__Chromerida	0.01%
d__Eukaryota;p__Chaetognatha	0.00%

Table S4 The taxon assignment of the contaminated gene profile in each metagenome

Taxon assignment	B1	B2	B3	B4	D1	D2	D3	D4	D5	D6
d__Eukaryota;p__Chordata	0.0040%	0.5214%	0.0038%	0.2156%	0.2156%	34.8501%	21.3673%	24.7156%	31.1030%	22.8450%
d__Eukaryota;p__Arthropoda	0.0003%	0.0005%	0.0001%	0.0014%	0.0014%	0.0103%	0.0060%	0.0020%	0.0085%	0.0030%
d__Eukaryota;p__Chlorophyta	0.0000%	0.0000%	0.0000%	0.0000%	0.0000%	0.0000%	0.0000%	0.0000%	0.0000%	0.0000%
d__Eukaryota;p__norank	0.3910%	0.3903%	0.6214%	0.1653%	0.1653%	0.6591%	0.4063%	0.4950%	0.6528%	0.4283%
d__Eukaryota;p__Streptophyta	0.0000%	0.0003%	0.0000%	0.0001%	0.0001%	0.0267%	0.0163%	0.0153%	0.0354%	0.0140%
d__Eukaryota;p__Cryptomycota	0.0424%	0.0509%	0.0352%	0.0539%	0.0539%	0.0012%	0.0011%	0.0030%	0.0043%	0.0015%
d__Eukaryota;p__Ascomycota	0.0621%	0.0681%	0.0897%	0.0451%	0.0451%	1.0782%	0.5575%	0.4153%	0.9575%	0.5555%
d__Eukaryota;p__Basidiomycota	0.0676%	0.0715%	0.1139%	0.0452%	0.0452%	0.3559%	0.2664%	0.1698%	0.3578%	0.2698%
d__Eukaryota;p__Microsporidia	0.0293%	0.1189%	0.0191%	0.0497%	0.0497%	4.9084%	4.1311%	5.1872%	4.5370%	3.6129%
d__Eukaryota;p__Mucoromycota	0.0373%	0.0498%	0.0419%	0.0444%	0.0444%	0.1538%	0.0952%	0.0740%	0.1817%	0.0965%
d__Eukaryota;p__Cnidaria	0.0324%	0.1135%	0.0592%	0.0622%	0.0622%	4.3196%	3.1710%	4.3739%	4.3417%	3.2172%
d__Eukaryota;p__Chytridiomycota	0.0241%	0.0253%	0.0341%	0.0190%	0.0190%	0.0000%	0.0000%	0.0000%	0.0000%	0.0000%
d__Eukaryota;p__Nematoda	0.0135%	0.0644%	0.0200%	0.0274%	0.0274%	2.2641%	1.8446%	1.9242%	2.2298%	1.6874%
d__Eukaryota;p__Zoopagomycota	0.0164%	0.0178%	0.0220%	0.0139%	0.0139%	0.0622%	0.0361%	0.0326%	0.0457%	0.0402%
d__Eukaryota;p__Mollusca	0.0241%	0.1513%	0.0370%	0.0881%	0.0881%	5.9992%	4.7266%	6.2146%	6.3359%	4.5324%
d__Eukaryota;p__Blastocladiomycota	0.0124%	0.0112%	0.0191%	0.0069%	0.0069%	0.0000%	0.0017%	0.0000%	0.0007%	0.0013%
d__Eukaryota;p__Echinodermata	0.0132%	0.1255%	0.0127%	0.0895%	0.0895%	5.8120%	4.2667%	5.5257%	5.4427%	4.2701%
d__Eukaryota;p__Platyhelminthes	0.0012%	0.0272%	0.0025%	0.0182%	0.0182%	1.8010%	1.7794%	4.1128%	2.4552%	1.8940%
d__Eukaryota;p__Euglenida	0.0005%	0.0006%	0.0059%	0.0050%	0.0050%	0.0000%	0.0000%	0.0000%	0.0000%	0.0000%
d__Eukaryota;p__Porifera	0.0044%	0.0085%	0.0032%	0.0156%	0.0156%	0.0771%	0.0282%	0.0370%	0.0633%	0.0477%
d__Eukaryota;p__Priapulida	0.0012%	0.0170%	0.0036%	0.0067%	0.0067%	0.4589%	0.5067%	0.5863%	0.6429%	0.4189%
d__Eukaryota;p__Bacillariophyta	0.0027%	0.0026%	0.0047%	0.0022%	0.0022%	0.0018%	0.0004%	0.0002%	0.0020%	0.0000%
d__Eukaryota;p__Apicomplexa	0.0018%	0.0038%	0.0026%	0.0029%	0.0029%	0.1114%	0.0600%	0.0984%	0.0426%	0.0351%
d__Eukaryota;p__Annelida	0.0016%	0.0027%	0.0039%	0.0016%	0.0016%	0.0064%	0.0064%	0.0135%	0.0041%	0.0197%
d__Eukaryota;p__Tardigrada	0.0023%	0.0014%	0.0025%	0.0008%	0.0008%	0.0000%	0.0000%	0.0000%	0.0000%	0.0000%
d__Eukaryota;p__Hemichordata	0.0012%	0.0027%	0.0027%	0.0017%	0.0017%	0.1258%	0.0856%	0.0703%	0.1844%	0.1230%
d__Eukaryota;p__Brachiopoda	0.0013%	0.0010%	0.0013%	0.0011%	0.0011%	0.0062%	0.0000%	0.0016%	0.0000%	0.0000%
d__Eukaryota;p__Placozoa	0.0030%	0.0028%	0.0059%	0.0010%	0.0010%	0.0000%	0.0000%	0.0000%	0.0000%	0.0000%
d__Eukaryota;p__Rotifera	0.0002%	0.0002%	0.0004%	0.0001%	0.0001%	0.0000%	0.0000%	0.0000%	0.0000%	0.0000%
d__Eukaryota;p__Chromerida	0.0008%	0.0004%	0.0011%	0.0004%	0.0004%	0.0000%	0.0000%	0.0000%	0.0000%	0.0000%
d__Eukaryota;p__Chaetognatha	0.0002%	0.0001%	0.0004%	0.0000%	0.0000%	0.0000%	0.0000%	0.0000%	0.0000%	0.0000%

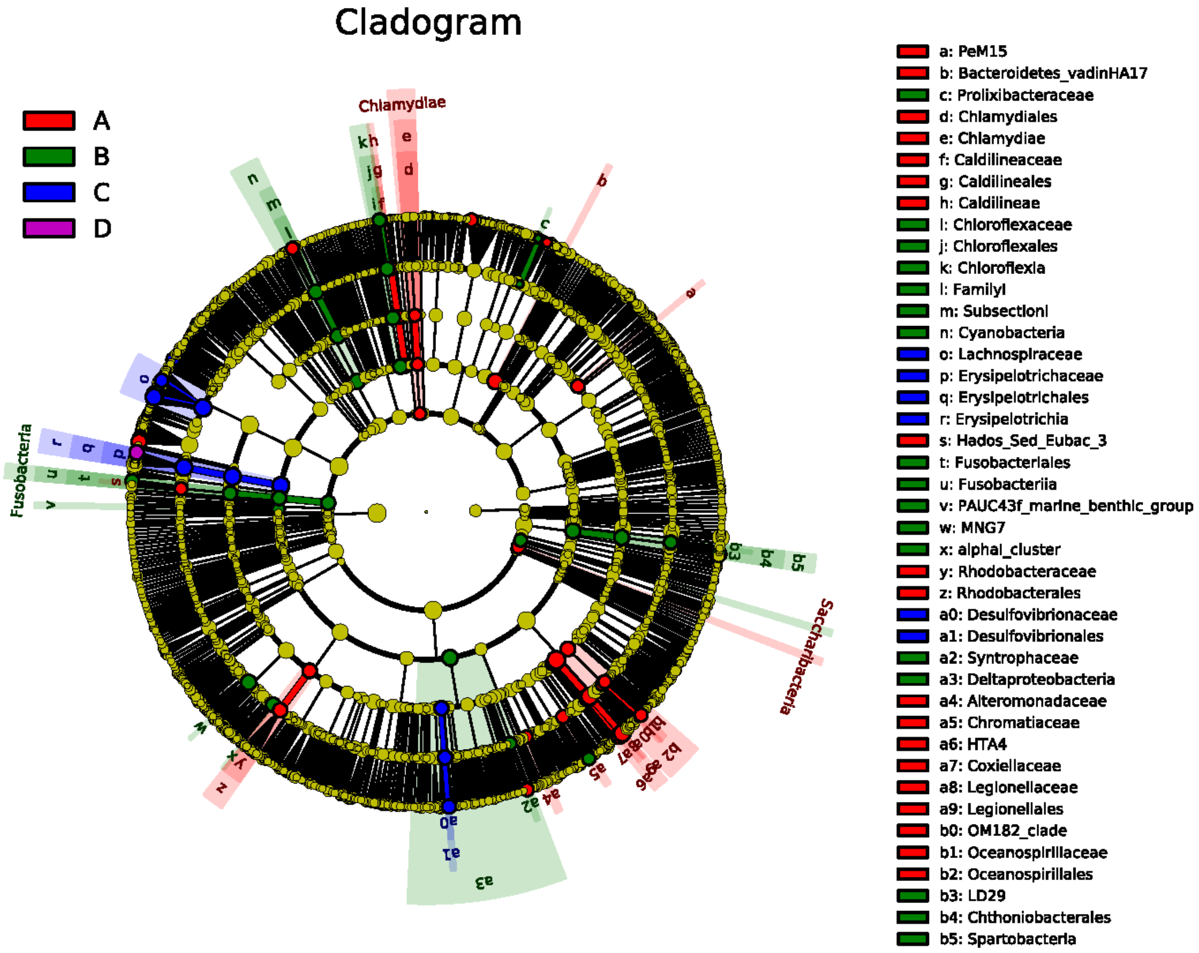

Figure S1 The LefSe analysis on the gut microbiomes among four groups using 16S rRNA gene sequences among four groups (Threshold on the logarithmic LDA score for discriminative features: at 3.0). A, MA group; B, MB group. C, MC group. D, MD group.

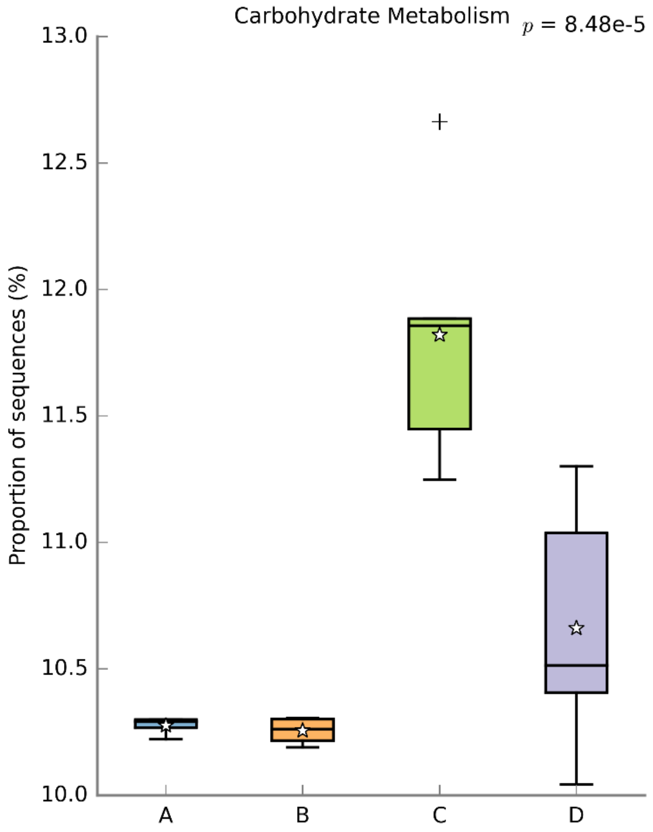

Figure S2 The predicted carbohydrate metabolism among four groups using 16S rRNA gene sequence. A, MA group; B, MB group. C, MC group. D, MD group.
